# Role of Fibroblast‐Immune Crosstalk in Kidney, Lung, and Skin Tertiary Lymphoid Structures

**DOI:** 10.1111/imr.70059

**Published:** 2025-08-29

**Authors:** Amy Cross, Jennifer Shelley, Rebecca Newman, Jessica Strid, Alice E. Denton

**Affiliations:** ^1^ Respiratory, Immunology & Inflammation Research Unit, GlaxoSmithKline Stevenage UK; ^2^ Department of Immunology and Inflammation Imperial College London London UK

**Keywords:** fibroblast, germinal center, kidney, lung, skin, tertiary lymphoid structure

## Abstract

Tertiary lymphoid structures (TLSs) are organized aggregates of lymphocytes, myeloid cells, and stromal cells that form at sites of inflammation, providing adaptive immune responses outside of secondary lymphoid organs (SLOs). Found in various pathological conditions—including chronic infections, cancer, organ transplantation, autoimmune diseases, and allergy—the presence of TLSs is linked to potentiation of local immunity. TLSs can be beneficial or detrimental, depending on context, and have been implicated as prognostic for disease severity and therapy response. Architecturally, TLSs resemble SLOs with distinct T and B cell areas supported by fibroblasts that secrete chemokines and cytokines that support immune cells. These structures must be created *de novo* in non‐lymphoid tissues; thus, the steps for TLS formation mimic, but do not completely copy, those of SLO formation. The accumulation of immune cells in tissues in inflammatory settings can initiate remodeling of tissue fibroblasts, leading to TLS formation; this process is common across tissues, although there are tissue‐ and disease‐specific pathways that impact TLS formation in certain contexts. This review will explore the immune‐stromal crosstalk in kidney, lung, and skin TLSs across a range of disease settings, highlighting shared as well as tissue‐specific mechanisms for TLS formation.

**Abbreviations**
APRIL: A proliferation‐inducing ligand
αSMA: alpha‐smooth muscle actin
BAFF: B cell activating factor
CCL: C‐C chemokine ligand
CCR: C‐C chemokine receptor
(c)SCC: (Cutaneous) squamous cell carcinoma
CXCL: C‐X‐C chemokine ligand
CXCR: C‐X‐C chemokine receptor
DC: dendritic cell
ECM: extracellular matrix
FDC: follicular dendritic cell
GC: germinal center
HEV: high endothelial venule
ICAM: intracellular adhesion molecule
IL: interleukin
ILC: innate lymphoid cell
(i)SALT: (Inducible) skin‐associated lymphatic tissue
LTi: lymphoid tissue inducer
LTo: lymphoid tissue organizer
LTβR: lymphotoxin‐beta receptor
MSC: mesenchymal stem cell
p75NTR: p75 neurotrophin receptor
PDGFR: platelet‐derived growth factor
polyI:C: polyinosinic:polycytidylic acid
RALDH2: retinaldehyde dehydrogenase 2
SLO: secondary lymphoid organ
TIRAP: TCDD‐inducible poly [ADP‐ribose] polymerase
TLS: tertiary lymphoid structure
VCAM: vascular cell adhesion molecule

## 1 Introduction

Tertiary lymphoid structures (TLSs) are organized aggregates of lymphocytes, myeloid cells, and stromal cells that provide sites for adaptive immune responses outside of secondary lymphoid organs (SLOs), which comprise the spleen, lymph nodes, tonsils, and Peyer's patches. TLSs originate as small clusters of B and T cells that can expand and mature into highly organized structures with germinal centers (GCs), specialized stromal cells including CD21+ follicular dendritic cells (FDCs), and high endothelial venules (HEVs) [1]. Mature TLSs anatomically resemble SLOs as complex aggregations of leukocytes supported by stromal cells; however, TLSs are not encapsulated and lack an independent vascular network. The lack of a capsule allows dendritic cells (DCs) to enter the TLS freely during maturation [2]. Functionally, TLSs can respond to locally presented antigens, including autoantigens in inflamed and damaged tissues in the context of autoimmunity and cancer. This results in lymphocyte proliferation, cytokine production, and local (auto‐)antibody production [3].

Unlike SLOs, TLSs require persistent inflammatory signals to form and are therefore often found at sites of chronic infection, cancer, organ transplantation, and autoimmune disease. The function of TLSs has garnered recent interest due to their association with disease severity, prognosis, and response to therapy [2, 4, 5]. The presence of TLSs is associated with a favorable disease course in cancers and infection but correlates with increased disease severity in the context of autoimmunity and chronic inflammatory disease [6].

Although the nomenclature for defining the different maturation stages of TLSs has been reported in different ways, there are common themes one can use to define them. Most published studies describe three distinct stages, with some defining the maturation groups as stage I, II, and III, whereas others refer to TLSs as early, primary, or secondary. The consensus is that stage I TLSs are composed of T and B cell aggregates, without GCs or FDCs; stage II TLSs lack GCs but contain a small B cell follicle and FDCs, and stage III TLSs contain prominent GCs, FDCs, HEVs, and specialized supportive fibroblasts [7, 8, 9, 10]. Fibroblasts are essential for SLO and TLS architecture, producing chemokines and cytokines that promote immune cell localization, survival, and differentiation. C‐C chemokine receptor (CCR)7‐expressing cells (T cells and DCs) are recruited toward C‐C chemokine ligand (CCL)19/CCL21, while C‐X‐C chemokine receptor (CXCR)5‐expressing B cells and GC‐like structures are supported by C‐X‐C chemokine ligand (CXCL)13‐expressing FDCs. TLS maturation is common between human and mouse, both mechanistically and phenotypically, indicating this is largely a conserved process between species [5], although the skin may be an exception as mouse skin is less susceptible than human to TLS formation.

Initial formation of stage I TLSs is characterized by immune cell infiltration, namely, CD3^+^ T cells and CD20^+^ B cells into non‐lymphoid tissue. This is facilitated by cytokine and chemokine release (e.g., CCL19, CCL21, CXCL13) by stromal cells. Stage I TLSs primarily contain proliferating cells and can be identified by Ki‐67 immunofluorescence staining [9, 11]. Maturation of stage I TLSs, by continued recruitment of immune cells and organization of B and T cells into distinct zones with the help of FDCs, gives rise to stage II TLS. FDCs also support B cell maturation and proliferation and at this stage, the TLSs start to become more vascularized, resulting in increased cell trafficking and retention within the tissue [3, 5]. Stage III TLSs are the most mature form of TLSs and are highly organized with defined B and T cell areas, functional GCs that enable B cell maturation, and production of high‐affinity antibodies and HEVs that maintain exchange of cells between the TLS and circulating blood. It is these mature TLSs that are responsible for formation of anti‐tumor antibodies in the case of some cancers and autoantibodies that correlate with disease severity in the context of autoimmunity [3, 5]. This review will focus on the role of stromal cells in TLS initiation, formation, and maturation in the lung, kidney, and skin.

## 2 Fibroblast Remodeling to Support Renal TLS

### 2.1 Structure of the Kidney

TLSs have been identified in the kidney in numerous renal pathologies, including chronic kidney disease, renal cell carcinoma, acute rejection of renal allografts, acute and chronic interstitial nephritis, lupus nephritis, and IgA and membranous nephropathy [12, 13, 14, 15, 16]. TLS formation in a murine acute kidney injury model has also been reported, where aged but not young mice exhibit multiple TLSs [17], suggesting TLS formation is common in a number of renal inflammatory settings. The following section of this review will focus on the role of stromal cells in TLS biology within the kidney of both human and mouse and discuss the similarities and differences in these processes between renal diseases, focusing on systemic lupus erythematosus/lupus nephritis, renal cell carcinoma, and kidney injury.

The kidneys are a pair of bean‐shaped organs situated in the retroperitoneal space. Their complex structure enables performance of vital functions; for example, the precise organization of blood vessels in the kidney allows filtration of large volumes of blood, facilitating reabsorption of vital nutrients, removal of waste products, and maintenance of homeostasis [18, 19]. The kidneys comprise an outer capsule, renal cortex, and inner medulla, which is further divided into renal pyramids. The medial side of the kidney contains the renal hilum, where the renal artery, renal vein, nerves, and ureter enter and exit the kidney; thus, the hilum connects the kidney to the circulatory and urinary systems [20]. Most nephrons, the functional unit of the kidney, span the renal medulla and cortex and consist of the glomerulus, Bowman's capsule, and convoluted tubules [20, 21].

Human and mouse kidneys share many fundamental features; however, human kidneys are much larger and contain significantly more nephrons. Furthermore, gene expression differences between mouse and human kidneys have been demonstrated in several publications [22, 23, 24]. A recent spatial omics comparison between mouse and human kidneys, enabling evaluation of gene expression without tissue dissociation, indicated that differentially expressed genes in mouse cortical regions were associated with energy production and metabolic processes [25]. While an increased metabolic rate in mouse tissues is a known phenomenon [24], the authors also highlighted that these differences may be attributable to age and environmental differences between their mouse and human samples. Despite this, the overlapping structure and function of kidneys between mouse and human make mice a good model organism for biomedical research [26].

### 2.2 Kidney Destruction in Disease

A high proportion of end‐stage kidney disease is caused by glomerular diseases which are exacerbated by the limited ability of the kidneys to undergo repair once damaged [18]. Kidneys are unable to generate new nephrons, resulting in a gradual decrease in their numbers over time, which may be further impacted by ongoing disease, injury, and aging [20]. In the aged kidney, both macroscopic and microscopic changes occur which alter the function of the kidney and increase susceptibility to developing renal pathologies such as chronic kidney disease and acute kidney injury. Examples of functional changes include reduced glomerular filtration rate and renal blood flow, overall reduction in kidney mass, and an increased presence of cysts and tumors, which potentially could lead to renal cell carcinoma [20, 27]. Autoimmune conditions can also lead to kidney damage, as is the case in systemic lupus erythematosus which can cause acute and chronic renal inflammation. Between 40 and 70% of systemic lupus erythematosus patients develop lupus nephritis, which is characterized by deposition of immune complexes in the glomeruli, leading to complement activation, further inflammation, and kidney damage [28].

### 2.3 What Are the Stromal Cells in the Kidney?

**FIGURE 1:**
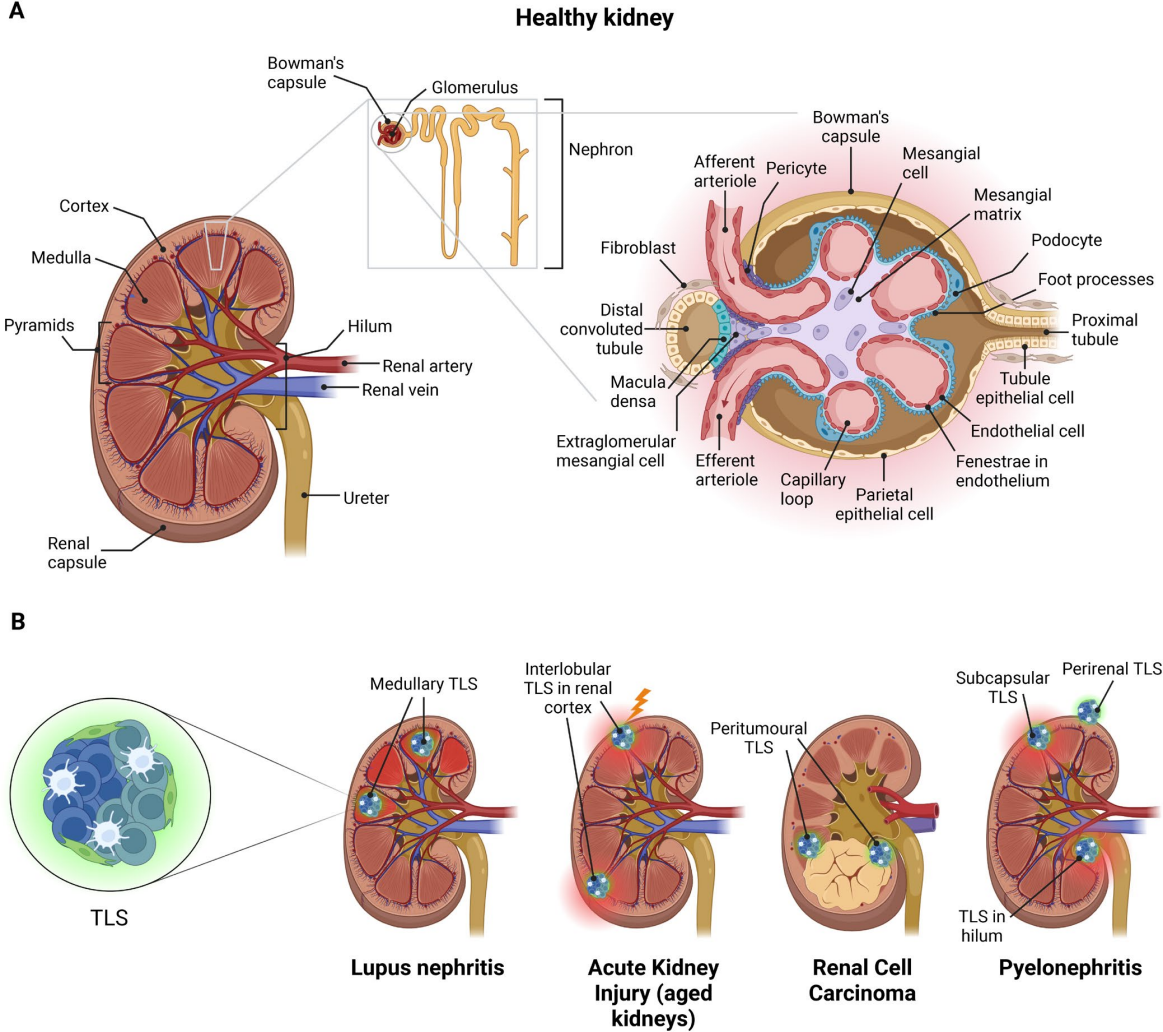
Normal kidney structure and TLS location in disease. Schematic showing: (A) the general structure of a healthy kidney depicting the gross anatomy of the kidney, the nephron, and the glomerulus, with the main tissue‐resident stromal cell populations. Note the absence of any immune aggregates in the healthy kidney; and (B) Four example scenarios where TLS have been observed in the kidney: Lupus Nephritis, acute kidney injury, renal cell carcinoma, and pyelonephritis with the described localization of TLS indicated.

Renal stromal cells are non‐epithelial, non‐endothelial cells that provide structural and functional support in the kidney. They originate from mesenchymal progenitor cells and differentiate into various cell types including pericytes, fibroblasts, endothelial cells, and mesangial cells (Figure 1A). This includes fibroblasts, which play an essential role in tissue structure and homeostasis under physiological conditions by supporting extracellular matrix (ECM) turnover through the production of ECM proteins such as collagen. In the kidney, fibroblasts provide structural support for the nephrons and directly interact with other kidney‐resident cells such as proximal tubular cells. They also play kidney‐specific functions such as the production of erythropoietin, a critical regulator of erythropoiesis, by a subset of cells found in the deep cortex and outer medulla in response to hypoxia [29]. Additionally, fibroblasts play a role in repair and regeneration processes following kidney injury; however, excessive activation can lead to transdifferentiation of fibroblasts into activated hypersynthetic fibroblasts, including myofibroblasts [30]. This leads to overproduction of ECM components, resulting in fibrosis and impaired kidney function [31, 32].

Pericytes are mural cells that surround vascular endothelial cells and control circulation. They share features with resident fibroblasts, including mesenchymal origin, morphology, and cellular markers such as platelet‐derived growth factor receptor (PDGFR)β [33], and are primarily distinguished based on anatomical location. Although predominantly considered vascular cells, renal endothelial cells, which line blood vessels including the glomerular capillaries, are specialized to maintain the filtration barrier and regulate ultrafiltration. Additionally, endothelial cells in the medulla maintain an osmolarity gradient and impact urine concentration [34].

Podocytes are terminally differentiated cells of the kidney glomerulus that are essential for the integrity of the filtration barrier enabled by their specialized structure, which includes foot processes [35]. Foot process effacement (loss of membrane extensions) is an early result of podocyte injury and is often accompanied by proteinuria. Early reversal of this structural change is essential to prevent ongoing damage and ultimately renal failure [35]. Mesangial cells are the glomerular stromal cells with similar properties to smooth muscle cells. They are located within the glomerulus where they provide structural support, produce and maintain the mesangial matrix, and regulate blood flow through their contractile capabilities [36, 37]. They also play a key role in clearing proteins and immune complexes from the glomerular basement membrane and can produce inflammatory cytokines when activated in response to injury [38].

### 2.4 Initiation of TLSs in the Kidney via Tissue Remodeling

TLSs are found in many areas of the renal parenchyma [9], with their location being context dependent (Figure 1B). In human and mouse aged and pyelonephritic kidneys, TLSs have been identified under the renal capsule, along the glomeruli, in the hilum and perirenal tissue [9]. More mature TLSs with distinct B and T cell areas are found in highly injured areas of the kidney, whereas TLSs are rarely found in mildly injured areas or in the medulla [9]. In contrast, TLSs have been observed in the medulla in lupus nephritis patients and lupus‐prone mice [39], whereas in a mouse model of acute kidney injury, TLSs are reported around intralobular and interlobular arteries in the renal cortex [11, 17]. In renal cell carcinoma, TLSs are primarily found at the tumor margin and within peri‐tumoral fibrotic and septal regions [10, 40, 41]. These areas are surrounded by blood and lymphatic vessels, providing a constant supply of circulating immune cells that supports TLS development [9, 17, 42].

Unlike SLO development, TLSs develop in the presence of lymphocytes. Further, TLSs form within inflamed tissues rather than as separate encapsulated organs, and in the context of chronic kidney diseases, this allows direct contact of the TLSs with the renal capsule [12]. Activation of the vasculature, including upregulation of adhesion and chemotactic molecules such as peripheral node addressin, mucosal addressin, cell adhesion molecule, intercellular adhesion molecule (ICAM)‐1 and CCL21, to drive lymphocyte recruitment is a prerequisite for TLS formation [43].

TLS formation in the adult shares some similarities with embryonic SLO development, which occurs at predefined anatomical locations resulting from stromal and endothelial organizer cells [44]; however, the chronology and molecular mechanisms underpinning TLS development have some notable differences. Unlike SLO formation, which occurs on the background of poorly differentiated mesenchyme, TLSs form in organs where tissue‐resident stromal cells are specialized to support local requirements. Therefore, stromal cell remodeling following a disturbance in tissue homeostasis is a prerequisite to TLS formation. TLS‐associated fibroblasts differentiate from postnatal, locally activated mesenchymal cells in the context of inflammation and chronic antigen presentation. In the kidney, resident fibroblasts demonstrate plasticity to respond to local cues and transdifferentiate into distinct fibroblast subtypes. Despite only forming a small component of TLSs, these differentiated fibroblasts play key roles in their initiation [7].

Sato et al. have used a model of ischemic reperfusion injury to characterize TLS formation in the kidney, which includes destruction of the renal parenchyma and healthy nephrons adjacent to the expanding TLS, and expression of proinflammatory cytokines which correlates with TLS size [11]. In the context of acute kidney injury, TLS formation has been shown to be initiated by a paracrine interaction between distinct fibroblast populations [11]. Perivascular fibroblasts receive retinoic acid signals from retinaldehyde dehydrogenase 2^+^ (RALDH2^+^) fibroblasts and dedifferentiate into p75 neurotrophin receptor (p75NTR ^+^) fibroblasts, a marker usually associated with neural crest cells. Some of these fibroblasts then acquire the ability to produce the chemokines CXCL13 and CCL19. This differs from SLO formation where CXCL13 is produced by lymphoid tissue organizer (LTo) cells induced by neuron‐derived retinoic acid [45]. p75NTR^+^ fibroblasts have also been shown to play a role in TLS initiation in patients with pyelonephritis and chronic kidney disease [9]. In the injury model, release of CXCL13 and other homeostatic chemokines such as CCL19 by the p75NTR^+^ fibroblasts leads to recruitment of immune cells into the TLS and formation of distinct B and T cell areas [11]. As injury develops, p75NTR^−^ areas within the TLS appear and CXCL13 starts to be produced by CD21^+^ stromal cells, indicating the development of FDCs. It is known that FDCs support GC responses, suggesting that the GC response is associated with later phases of TLS formation. CCL21 is another chemokine known to play a crucial role in SLO development and maintenance; however, Sato et al. [11] concluded that CCL21 is not involved in age‐associated TLS formation in the kidney based on weak correlation of CCL21 expression with TLS size.

TLSs predominantly form proximally to vascular or epithelial duct structures that sit next to pericytes, smooth muscle cells, or myofibroblasts, which share functional and phenotypic features of LTo cells [46, 47, 48]. Combined expression of lymphotoxin (LT)α and β drives the formation of highly organized lymphoid structures [49] but is not absolutely required to prime the stromal cell compartment ahead of TLS formation. Leukocytes, including myeloid cells and granulocytes, which are abundant in the earliest phases of inflammation, can drive the initiation of TLSs through the release of proinflammatory cytokines, resulting in the activation of resident fibroblasts [50]. Following tumor necrosis factor (TNF) receptor engagement, a population of α‐smooth muscle actin (αSMA)^+^ Podoplanin^+^ fibroblasts can differentiate in non‐lymphoid tissue during inflammation and cancer and express lymphoid chemokines, including CXCL13, CCL21, CCL19, and CXCL12, as well as lymphocyte survival factors such as IL‐7, B cell activating factor (BAFF) and A proliferation‐inducing ligand (APRIL), which are involved in TLS establishment and maintenance [46, 51, 52]. The production of cytokines that drive stromal cell activation by leukocytes other than lymphocytes indicates that this may occur prior to and independently from lymphocyte accumulation [48].

Mesenchymal stem cells (MSCs) may also play a key role in TLS initiation in kidney pathologies, as shown by Dorraji et al. in a study investigating the link between interleukin (IL)‐1β and TLS formation in lupus nephritis patients and lupus‐prone mice [53]. When stimulated with interleukin (IL)‐1β, human MSCs upregulate their expression of CXCL13, CCL19, ICAM‐1, and vascular cell adhesion molecule (VCAM)‐1 and act as LTo cells, activating CD4^+^ T cells. Murine MSCs were observed in the pelvic wall and kidney TLSs, and similar gene expression profiles were seen compared with humans. These tissue‐specific MSCs therefore could play a key role in driving inflammation and initiating TLS formation in lupus‐prone mice and lupus nephritis patients; however, tissue‐specific depletion of MSCs would be required to confirm this hypothesis [53].

Kidney‐resident cells in lupus nephritis patients, such as renal tubular epithelial cells, mesangial cells, and podocytes, are also involved in the initiation of renal TLSs through the production of various cytokines, leading to further damage and injury to the kidney [39]. In lupus‐prone mice, tubular epithelial cells enhance the recruitment of lymphocytes to the kidney interstitium via their expression of IL‐23 receptors and the production of CCL20. Upon binding of IL‐23 to tubular epithelial cells, calcium/calmodulin‐dependent protein kinase IV expression increases, which halts the production of arginase 1 and leads to the accumulation of arginine in the kidney, supporting lymphocyte expansion [54].

Overall, this demonstrates a complex interplay between a range of kidney‐resident stromal cells in driving the initiation of TLS through recruitment of leukocytes into the kidney.

### 2.5 TLS Maturation

In chronic inflammatory environments, TLS progression and maturation correlate with the severity of disease and the extent of kidney damage. This correlation has been shown in kidney injury in mice [11], as well as in aged pyelonephritis patients [9], and patients with lupus nephritis, IgA nephropathy, and transplanted kidneys [5]. Contrastingly, the presence and maturation of TLS in many cancer types, including renal cell carcinoma, correlate with better survival and response to treatment [2].

As outlined above, kidney‐resident stromal cells play an important role in the formation and maintenance of TLS in the kidney in both humans and mice, and this occurs by a positive feedback mechanism. Continuous interactions between immune cells and fibroblasts increase the production of chemokines and cytokines, which supports the TLS environment and facilitates further immune interactions [55]. CD11b^+^ myeloid cells have been reported to secrete BAFF, a key B cell survival factor, which enhances B cell chemotaxis into the kidney, activates local T cells, and leads to further aggregation and compartmentalization of TLSs [56, 57]. Maturation of TLSs in the kidney comprises several distinct stages, which reflect the extent of organization and functional abilities of the TLS. The dynamic interaction between kidney‐resident stromal cells and infiltrating immune cells is reflected in the maturation stages, which were outlined previously in this review [5].

### 2.6 Comparison of TLS Initiation and Maturation Between Different Renal Pathologies in Human and Mouse

TLSs have been identified in many renal pathologies, such as systemic lupus erythematosus, lupus nephritis, chronic kidney disease, acute kidney injury, renal cell carcinoma and their formation, and their maturation and potential roles in disease have been studied in human and mouse. Mechanistic studies have shown that TLS formation in human and murine kidneys is driven by common fibroblast‐chemokine pathways. For example, resident fibroblasts are responsible for TLS initiation in both species, specifically RALDH2^+^ and p75NTR^+^ fibroblasts, and the production of CXCL13 and CCL19 plays a key role in TLS formation in both species. TLS maturation thus follows a similar mechanism in human and murine kidneys [11, 33, 58].

TLSs have been identified in aged, but not young human kidneys, correlating with the ability of young but not elderly patients to recover from acute kidney injury [11]. This observation is also true in mice as TLSs have only been identified in the kidney of aged mice after AKI but were absent from the young controls [8, 11]. While there is considerable overlap in the cellular and molecular aspects of TLSs in aged human and murine kidneys, the temporal kinetics of TLS formation differ between species, with TLSs forming quicker in murine compared to human kidneys [11].

TLS architecture, chemokine expression, and resident fibroblast involvement are similar between different human renal pathologies, indicating that TLS formation is organ‐specific rather than disease‐specific. However, the function of TLSs and their impact on disease differ between renal cell carcinoma and systemic lupus erythematosus/lupus nephritis, chronic kidney disease, and acute kidney injury. In the context of systemic lupus erythematosus/lupus nephritis, chronic kidney disease, and acute kidney injury, TLSs are linked to disease progression, autoantibody production, and exacerbation of injury; blocking TLS has positive outcomes, such as reducing damage to the kidney [3, 11, 55]. This suggests that TLSs could be an attractive therapeutic target through disruption of cell–cell interactions, key in TLS initiation, or by targeting survival factors required to sustain TLSs. Indeed, TNFR superfamily member 8 (CD30)/CD153 signaling between CD153+ senescence‐associated T cells and CD30+ age‐associated B cells has been implicated in TLS expansion in aged murine kidneys after injury [59], and elevated circulating CD30 levels correlate with the presence of TLS and progression of IgA nephropathy [60]. Deficiency of either CD153 or CD30 attenuated TLS formation, resulting in reduced inflammation and fibrosis and improved kidney function [59]. BAFF, another member of the TNF superfamily, is upregulated in the kidneys of lupus nephritis patients and mouse models, where it promotes the formation and survival of renal TLSs [61, 62]. Blockade of BAFF in mice disrupts TLSs and ameliorates nephritis [61], while in humans, BAFF expression correlates with TLS‐like B cell aggregates and disease severity [62, 63], suggesting potential therapeutic benefit from targeting BAFF in lupus nephritis. In contrast, in the context of renal cell carcinoma, renal TLSs promote B cell maturation towards plasma cells, provide a local source for anti‐tumor antibodies, and show an association with improved clinical outcomes and response to immunotherapy [64].

## 3 TLSs in the Lung

### 3.1 Introduction to the Lung

**FIGURE 2:**
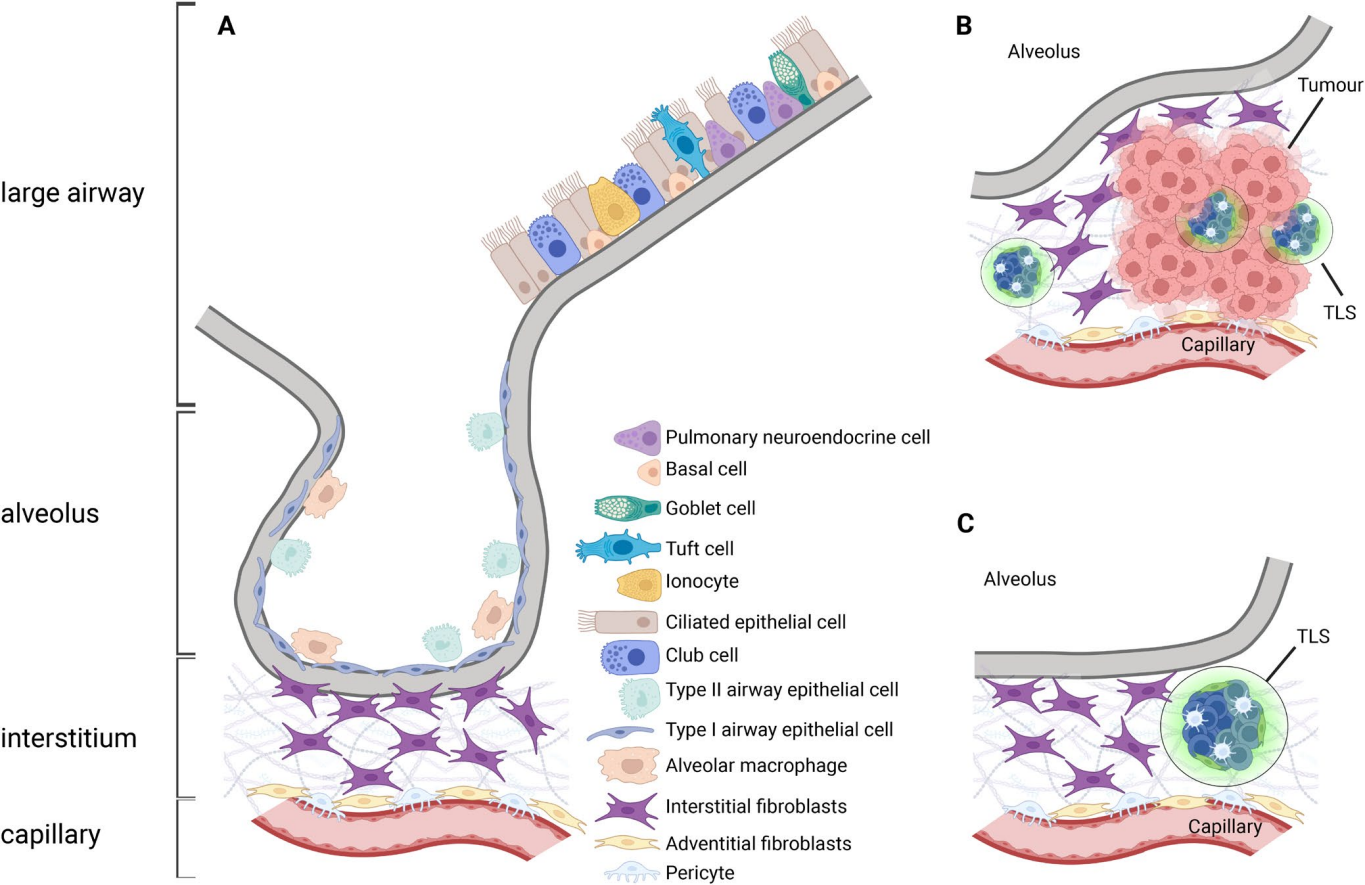
Stromal architecture of the lung and TLS formation. Schematic showing: (A) the structure of the lung, depicting the upper airways and the lower alveolus with adventitial and interstitial fibroblasts depicted; and examples of pulmonary TLS formation in cancer (B), where TLSs can form within tumors, at the tumor margin, or in the interstitial space; or in infection (C), where TLSs typically form between the bronchus and blood vessels.

The lung is comprised of two bronchi (left and right), which extend from the trachea; each bronchus branches into smaller bronchioles in each lobe (Figure 2A). The bronchioles end in small air sacs called alveoli, where gas exchange occurs—the central function of the lung. The lung microarchitecture is comprised of a range of cell types that support specific functions in different parts of the lung [65]; the cellular composition adapts as the lung develops during embryogenesis and in response to altered pulmonary conditions such as infection or inflammation.

Because the lung is constantly exposed to the external environment, it must also act as a barrier to potential pathogens. Unlike other mucosal surfaces, such as the gut, the lung does not have a defined constitutive mucosal‐associated lymphoid tissue. The healthy lung is typically devoid of lymphoid aggregates in mouse and human [66], although rats and rabbits do show evidence of lymphocyte aggregates in the healthy lung [67]. Pulmonary TLSs typically form adjacent to bronchioles (Figure 2B), between the airway and a blood vessel, and have been termed inducible (in humans/mice) bronchus‐associated lymphoid tissue. For continuity in this review, they will be herein referred to as pulmonary TLSs.

### 3.2 Pulmonary TLSs

Pulmonary TLSs form in a range of diseases, including cancer, infection, autoimmune disease, as well as in chronic inflammatory diseases. The presence of TLSs is thought to potentiate local immune responses, exacerbating local disease in the case of inflammatory and autoimmune diseases, or enhancing immune‐mediated clearance of pathogenic material in the case of cancer and infections. In the context of cancer, there is a strong correlation between the presence of mature GC‐containing TLSs and better outcomes from cancer [68, 69, 70], and the response to immune checkpoint blockade [71]. In the context of infection, TLS formation has been investigated largely in mouse models, owing to the difficulty of sampling human lung tissue. TLS formation is observed in several respiratory infections, including influenza A virus [72, 73, 74], *M. tuberculosis* [75, 76, 77], modified vaccinia Ankara [78], and Pneumocystis [79], among other infections. In chronic inflammatory conditions such as asthma, chronic obstructive pulmonary disease, and idiopathic pulmonary fibrosis, increasing severity of disease correlates with pulmonary TLS formation [6].

#### 3.2.1 Fibroblasts in the Lung

The healthy lung has a diverse population of mesenchymal cells, with different regions of the lung supported by specialized fibroblasts, typically identified by their interstitial or adventitial location. Adventitial fibroblasts are adjacent to blood vessels and support blood vessel structure and function, while interstitial fibroblasts are larger, stellate, and synthesize more collagen [80]. While there is little turnover of lung fibroblasts in the steady state, any damage to the lung must be repaired to allow continued gas exchange, and the alveolus also acts as a stem cell‐supportive niche in which PDGFRα+ fibroblasts react to tissue damage to coordinate repair [81]. The highly vascularized surface of the lung is also supported by pericytes, which ensheathe capillaries and can be in direct contact with endothelial cells [82]. Because pericytes share cell surface markers with fibroblasts, such as PDGFRs, the distinction between these two cell types in the lung usually requires spatial information: fibroblasts are within the interstitium or adventitia, while pericytes are distinctly perivascular. Recent advances in single cell and spatial sequencing methods have further defined the complexity of lung cells, including fibroblasts [65, 83, 84, 85], and the data suggest that specific fibroblast types are associated with immune cell niches in the lung.

Immunomodulatory fibroblasts are not typically present in the healthy lung; their development is thought to occur through differentiation of local mesenchymal cells that acquire immunomodulatory functions. Both pericytes and fibroblasts can differentiate into SLO‐like fibroblasts that typically form the stromal basis of a TLS. In vivo experiments with lineage‐tracing mouse models have shown that TLSs are derived from local progenitors [86], with both pericytes and adventitial fibroblasts able to give rise to TLS‐supportive fibroblasts [87]. Intriguingly, the latter study suggested that pulmonary TLSs can have different origins and transcriptional profiles: CCL19+ TLS fibroblasts resembling peptidase inhibitor 16 (Pi16) + SLO fibroblasts [88, 89] seemed to be pericyte‐derived, while CCL19+ fibroblasts that resemble T zone SLO fibroblasts [90] were derived from adventitial progenitors [87]. Mouse lineage‐tracing models implicated local differentiation, with the fibroblasts in each TLS likely deriving from a common progenitor, although there is a dearth of data exploring TLS fibroblast origins and functions and the immunological impact thereof.

### 3.3 Pulmonary TLS Formation

The recruitment of T and B cells is the essential requirement for pulmonary TLSs. Once in the lung, T and B cells can differentiate into GC‐like and follicular helper T cell‐like cells that then form a GC locally, and this can occur directly in the lung [91, 92]. This means that naïve T and B cells can be recruited directly into the lung from the blood—without need for prior activation in the lymph node [91]. Inflammation, alongside antigenic stimulation, is essential for pulmonary TLS formation, with TLR signaling, type I IFNs, and cytokines such as IL‐1α and/or IL‐17 shown to promote pulmonary TLS formation. Pulmonary TLSs are a distinct structure, perhaps best exemplified in pulmonary *M. tuberculosis* infection, where T cell and macrophage‐rich granulomas form, which are essential to contain the bacterium and limit spreading. TLSs are situated adjacent to, but distinct from, these granulomas [77] and are distinguished by their enrichment of B cells.

#### 3.3.1 Chemokines Underpinning Pulmonary TLSs

TLS formation depends on the generation of chemokine gradients that mimic those of SLOs, and the essential chemokine underpinning a specific TLS is context dependent. For example, heat‐killed *Pseudomonas aeruginosa* induces CXCL12‐dependent TLSs, while modified vaccinia Ankara‐induced TLSs are CXCL13‐based [78]. In comparison, influenza A virus‐induced TLSs are CXCL13‐ and CCL19/21‐supported [73, 92]. The essential requirement for CXCL13 or CCL19/21 in influenza‐induced pulmonary TLSs has been dissected in splenectomized mice lacking CXCL13 and/or CCL19/21 (paucity of lymph node T cells, plt/plt) [92]. In this model, T and B cells can only be primed locally in the lung, meaning the immune response is SLO‐independent. The lack of CXCL13 alone does not affect pulmonary B cell responses, as GC‐like cells were detected; however, TLS architecture was disrupted: CXCL13‐deficient mice have dispersed B cell aggregates with a complete lack of FDCs that would support a local GC; despite this, the GC is functional as antibody production is not affected. CCL19/21‐deficient animals retain GC‐like TLS structures with associated FDCs but have impaired GC B cell numbers and antibody output, perhaps due to impaired pulmonary T cell responses, while the absence of both CXCL13 and CCL19/21 abrogates TLS formation entirely. CXCL13‐ or CCL19/21‐dependent stromal cell networks that support B cell clustering and GC‐containing TLS formation are also essential for allergy‐induced TLSs [93, 94]. Thus, the central defining characteristic of pulmonary TLS—SLO‐like chemokine hubs for lymphoid aggregation—is largely agnostic to the disease type, although specific inductive pathways and immunological functions are likely to be context‐specific. Whether the structure of a TLS impacts its function remains to be determined, although this has been suggested in lung cancer [87].

#### 3.3.2 Drivers of Chemokine Expression in the Lung

Lymphotoxin‐beta receptor (LTβR) and TNF are key signaling molecules that lead to CXCL13 and CCL19/21 expression in SLOs [95]; however, they are not essential for pulmonary TLS formation. LTα‐deficient mice are able to generate delayed protective immune responses to influenza [73, 96] and murine gamma herpesvirus 68 [97] infections, suggesting functional TLS formation in the absence of LT/LTβR signaling. Indeed, mice deficient in both LTα and TNF express CXCL13 in the lung in response to influenza infection, despite no expression in SLOs. TLS structure was, however, affected, comprising loose lymphocytic aggregates consisting mostly of B cells—T cell recruitment was scant, suggesting poor CCL19/21 induction. While this might suggest TLS formation is independent of LTβR signaling, these data mimic those observed in mice in which LTβR expression was abrogated specifically in SLO fibroblasts [98]. While lymph nodes formed normally, they were smaller, and SLO fibroblasts were phenotypically immature and had significantly lower expression of CCL19/21, and CXCL13 was not affected. These functional differences were associated with impaired, but not abrogated, T cell immunity, suggesting that the same—albeit complex—‘rules’ that govern chemokine induction in SLOs are likely also occurring in pulmonary TLSs.

The induction of TLS‐supportive chemokines is context dependent and can be driven by a number of inflammatory pathways. Local inflammation is essential, as the presence of lung TLSs correlates with lung damage in systemic chronic diseases such as rheumatoid arthritis and Sjögren's syndrome patients with pulmonary symptoms [99]. IL‐17 has been implicated in many different TLS‐inducing conditions, supported by other cytokines that synergize to promote CXCL13 induction. Heat‐killed *P. aeruginosa* ‐induced CXCL12 requires IL‐17 signaling, while modified vaccinia Ankara‐induced TLSs are CXCL13‐based and do not require IL‐17 [78]. In comparison, influenza infection induces CXCL13 independently of IL‐17 in the adult [100] but seems to require IL‐17 in the neonatal lung [101]. Pneumocystis infection also induces TLSs via IL‐17 signaling, which synergizes with type 2 helper T cell‐derived IL‐13 to promote CXCL13 expression [79]. IL‐17 is also essential for *M. tuberculosis* ‐induced pulmonary TLSs, where IL‐17 expression is sustained by IL‐23; IL‐23‐deficient mice have fewer individual TLSs that are smaller, linked to poor CXCL13 expression [102]. In this model, IL‐23 was essential for long‐term TLS maintenance and anti‐ *M. tuberculosis* immunity, with IL‐22 and IL‐17 also contributing to CXCL13 maintenance.

The innate inflammatory mediators, type I interferon (IFN) and IL‐1, have both been implicated in pulmonary TLS formation during viral infection. Type I IFNs are expressed in response to viral infection and establish an antiviral state in neighboring cells to limit viral replication [103]. Type I IFN is highly induced in influenza infection and can directly induce *Cxcl13* expression by primary lung fibroblasts in vitro [72]. In fact, delivery and/or induction of type I IFN alongside a model antigen can induce TLSs with antigen‐specific GC B cells in the lung [72], suggesting this is a key pathway for pulmonary TLS formation. A role for the type I IFN pathway is supported in other models. Mutations in the type I IFN regulatory protein TCDD‐inducible poly [ADP‐ribose] polymerase (TIRAP) are associated with increased tonic type I IFN production; mice lacking TIRAP or its catalytic function have spontaneous pulmonary TLSs, as well as TLSs in other tissues, suggesting the type I IFN pathway for TLS induction is not unique to the lung. Sustained type I IFN is required for pulmonary TLS formation however, as delivery of a single dose of polyinosinic:polycytidylic acid (polyI:C) can induce pulmonary *Cxcl13* but not TLSs; TLSs require repeated polyI:C dosing for development [104]. Mechanistically, type I IFN promotes CXCL13 expression via upregulation of LTβR on pulmonary fibroblasts [104]. Intriguingly, the type I IFN‐LTβR signaling axis did not affect CCL19 induction in the lung, and depletion of CCL19‐expressing cells did not affect TLS formation in this polyI:C inflammation model, suggesting these cytokines are regulated by different pathways.

IL‐1R signaling is another early inflammatory mediator produced by epithelial cells and macrophages following viral infection [105]. IL‐1α acts in inflamed tissues to induce expression of molecules that promote leukocyte recruitment to the tissue, including chemokines (e.g., CXCL8) and adhesion molecules. IL‐1R signaling also contributes to pulmonary TLS formation: Il1r1‐deficient mice have delayed viral clearance and immune responses; despite this enhanced inflammatory state, they do not generate TLSs—CXCL13 is poorly induced, although CCL19/21 induction is not affected, and pulmonary GCs are not detected [106]. IL‐1R signaling in radioresistant cells is the key driver, suggesting that directly signaling through IL1R in fibroblasts drives this response. IL1R signaling is also important for the generation of TLSs during allergic inflammation, where particulate adjuvants delivered intratracheally cause alveolar macrophage death, releasing IL‐1α that promotes TLS formation. IL‐1 and IL‐17 have also been implicated in TLS formation in chronic obstructive pulmonary disease [107]; thus, the link between these signaling pathways and pulmonary TLS formation is not unique to viral infections.

Immune and non‐immune cells can act as sources of chemokine‐inducing cytokines or signaling molecules. LTβR signaling is a key regulator of fibroblast differentiation, and its ligand, LTαβ, is widely expressed by immune cells including T cells, B cells, and DCs. Since CXCL13 and CCL19/21 induction precede T and B cell infiltration, partly because B cell infiltration is dependent on CXCL13 induction [72], these cells are unlikely essential sources of LT signals. DCs are a key source of LTαβ for pulmonary TLS maintenance as their depletion after TLS formation abrogates TLSs and impairs lung GCs and IgA production [108]. A recent study showed that leukemia inhibitory factor inhibits TLS formation [109], and leukemia inhibitory factor appears to impact a number of TLS‐initiating pathways. Leukemia inhibitory factor‐deficient animals have increased retention of CCR7+ cells in the lung, particularly plasmacytoid DCs, which also have enhanced production of type I IFNs in the absence of this protein. Leukemia inhibitory factor also promotes CCL21 expression by lymphatic endothelium in the lung, thereby promoting emigration of CCR7+ cells from the lung. Thus, the combination of increased type I IFN and increased CCR7+ DCs in the lung likely synergizes to significantly increase pulmonary TLS formation in both infection and allergic inflammation models.

The cellular drivers of TLS formation are linked to those that produce inflammatory mediators that drive CXCL13 induction, such as type 3 innate lymphoid cells (ILC3), lymphoid tissue inducer (LTi) cells (a subset of ILC3s), γδ T cells, and type 2 ILCs, a lung‐specific subset of ILCs whose expression of leukemia inhibitory factor in response to infection or inflammation influences TLS formation, as outlined in the previous section. TLS‐associated ILC3 have been described in human lung cancer [110], where they express IL‐22, TNF, IL‐18, and IL‐2. Co‐culture of fibroblasts with tumor‐derived ILC3s induced upregulation of ICAM‐1 and VCAM‐1 expression by fibroblasts, suggesting the ILC3 can promote a TLS‐supportive fibroblast phenotype. ILC3 can also act as a key source of IL‐22 and an essential cellular driver of TLS formation in a lung allograft model [111]. LTi cells, which are essential for SLO formation via provision of LT signaling during embryogenesis, may also contribute to TLS formation. Forced retention of LTi cells at non‐SLO sites during embryogenesis leads to ectopic lymphoid aggregate formation [112]. LTi cells are found in the lung, but they are not required for pulmonary TLS formation [101]. It is important to note that LTi cells are an essential source of LT during embryogenesis, which occurs prior to T and B cell production. They are perhaps not required during ectopic TLS formation, where other immune cells can provide initiator signals such as LT, IL‐17, and/or IL‐22 [113].

### 3.4 Immunological Functions of Pulmonary TLSs

The true immunological impact of TLSs is difficult to unpick from the SLO‐mediated immune response, although mouse models and bioinformatic approaches have made significant progress on this recently. The presence of TLSs in cancer is strongly associated with improved outcomes, i.e., long‐term survival or disease‐free progression, as well as better responses to immune checkpoint blockade [114]. Lung cancer‐associated TLSs have variable immune components and can include GC responses in which high‐affinity antibodies are generated locally in the tissue as well as GC‐independent T cell‐mediated responses; importantly, the presence of mature TLSs is associated with improved responses to immune checkpoint blockade [71, 115]. In non‐small cell lung carcinoma, pulmonary TLSs within the same lung have functionally distinct TLS fibroblast networks that have immunological implications. TLSs supported by CCL19+ TRC‐like fibroblasts were closely associated with CD8+ T cells, and depletion of CCL19+ pulmonary fibroblasts led to enhanced tumor growth through dampened T cell immunity [87], suggesting TLSs specifically contribute to local CD8+ T cell‐mediated anti‐tumor immunity. The role of TLS immune responses in immune checkpoint blockade efficacy is emphasized by a recent study investigating why the response to immune checkpoint blockade is variable among non‐small cell lung carcinoma patients with mature TLSs. In this study, immune checkpoint blockade failure is associated with suppressive cancer‐associated fibroblasts [71]. Two subsets of suppressive cancer‐associated fibroblasts were identified: Fibroblast activation protein‐α + cancer‐associated fibroblasts were found to promote the inflammatory response, immune exclusion, and CD8+ T cell exhaustion, while MYH11+ cancer‐associated fibroblasts created an immunosuppressive environment through recruitment of regulatory T cells, competing with the TLS‐mediated enhancement of the anti‐tumor response. This emphasizes the importance of considering fibroblast diversity and its influence on tissue immunity.

GC‐containing pulmonary TLSs are also associated with improved outcomes and response to immune checkpoint blockade [116]. GC formation is associated with high cellular density in lung squamous cell carcinoma‐associated TLSs [70]. In this study, GC‐containing TLSs correlated with better outcomes in untreated patients; however, those treated with corticosteroids lose TLSs in the tumor alongside the predictive association of TLSs. In lung adenocarcinoma, pulmonary TLSs that contain GCs are CXCL13‐dependent and generate cancer‐specific antibodies against endogenous retroviruses that are expressed by the cancerous cells [117]. High‐affinity antibodies are generated in lung TLSs, and the pulmonary TLS is responsive to immunotherapy. Immune checkpoint blockade (anti‐PD‐L1) increases the lung GC response and improves survival; better survival may be due to enhanced NK cell‐mediated antibody‐dependent cell cytotoxicity. The immunological value of lung GC‐containing TLSs has been extensively evaluated in influenza infection, using SLO GC B cells as a comparator. Adachi et al. observed increased cross‐reactivity in lung‐derived GC B cells, compared to those from the lymph node [118]; subsequent studies demonstrated that this was due to differing antigenic epitopes in each tissue—a cryptic epitope in the haemagglutinin protein is exposed during fusion in the lung, and systemic administration of an engineered form of this antigen induced cross‐reactive responses in the lymph node [119]. Despite strong evidence for selection occurring in ectopic GCs, evaluation of the efficacy or quality of this response has been, to date, quite difficult. A recent study [91] used a model of house dust mite‐mediated inflammation combined with the model antigen hydroxy‐3‐nitrophenyl acetyl [120] to dissect the kinetics and function of lung and lymph node GC responses. Lung GC formation was delayed relative to the lymph node, and this was associated with a lower rate of somatic hypermutation and affinity maturation. At later stages, when the GC is fully established, there was no discernible difference in the quality of the pulmonary TLS‐derived GC response: the GC was structurally similar, and somatic hypermutation and affinity in lung GC B cells were equivalent to those observed in lymph nodes. Importantly, efficient somatic hypermutation and affinity maturation were also observed in pulmonary GC‐derived memory B cells [91]; thus, pulmonary GCs can generate high‐quality local immunity.

TLS‐derived memory B cells are long‐term residents in the lung—upon re‐infection they are quickly mobilized to the infected area and differentiate into plasma cells, providing a rapid early source of antibody that limits secondary infection [121]. This rapid reaction occurs adjacent to infected alveoli, suggesting that memory B and plasma cells occupy a non‐TLS niche. Indeed, a plasma‐cell‐specific niche has been described in the submucosal gland [65], where serous cells expressing CCL28, IL‐6, and APRIL are associated with CCR10‐expressing lung plasma cells. Memory CD4+ T cells were also in this submucosal gland niche, associated with epithelial cells expressing high levels of MHC II, suggesting they are positioned to rapidly respond upon re‐infection. The ability of the lung to support long‐term local memory cells that can rapidly respond upon infection prompts one to consider how they may be exploited therapeutically. Pediatric lungs have a high frequency of pulmonary TLSs with functional GCs, a life stage at which SLO GCs are inefficient, suggesting pulmonary TLSs may provide essential immunity early in life [122]. Repeated administration of lipopolysaccharide to neonatal mice over the first week of life induces mature pulmonary TLSs upon subsequent exposure to inflammation [101]. The TLSs induced by neonatal lipopolysaccharide exposure were more mature than those observed in adults, with high‐density T and B cells and clear FDC networks. Why the neonatal lung is more permissive to TLS formation is not clear—perhaps neonatal lung fibroblasts are more plastic than those present in adults, more readily acquiring an SLO‐like phenotype in response to inflammation.

## 4 TLS Formation in the Skin

### 4.1 Structure of Healthy Skin

**FIGURE 3:**
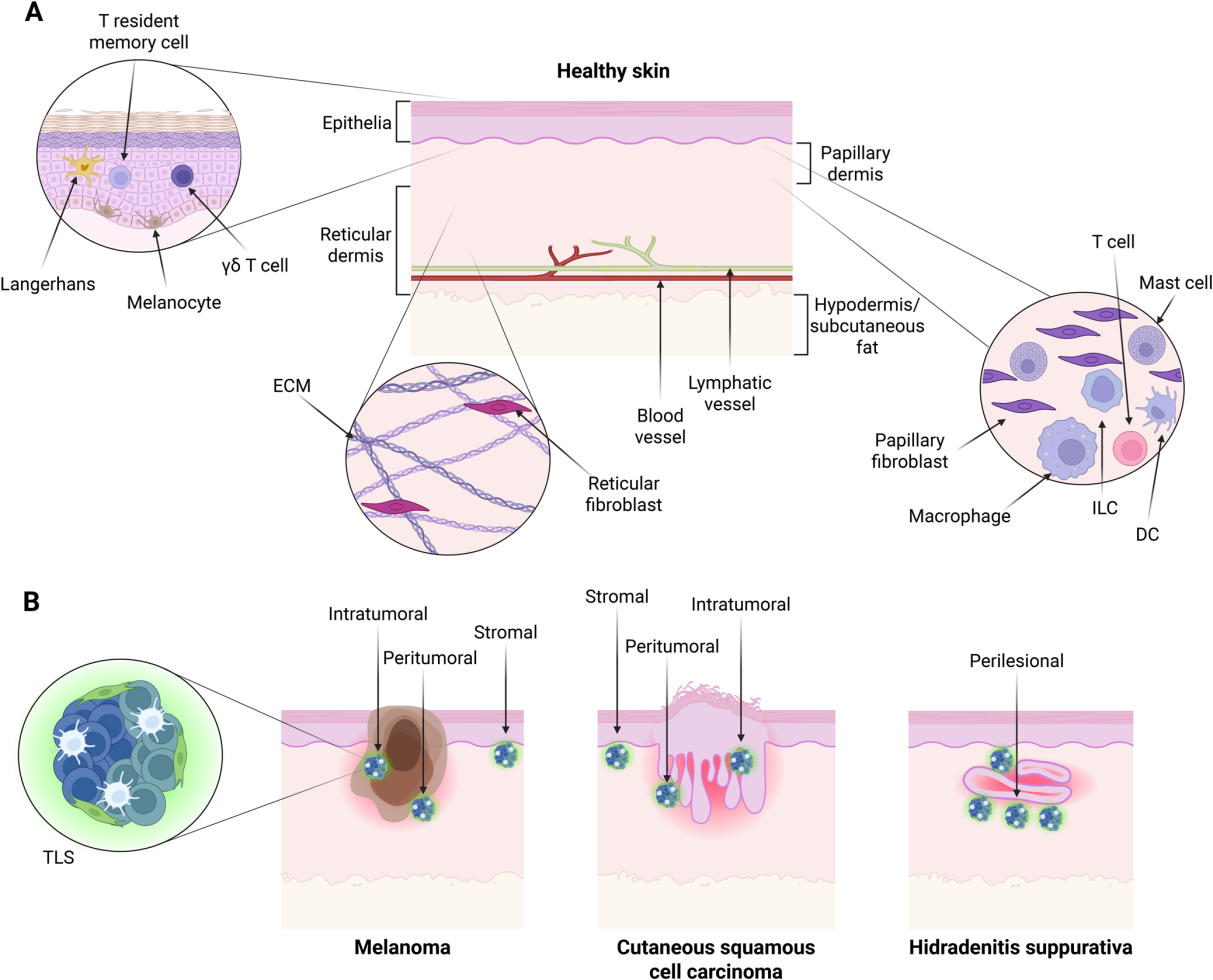
Skin architecture and TLS location in disease. Schematic showing: (A) the general structure of healthy skin with the 3 main layers: epidermis, dermis, and hypodermis depicted with the main tissue‐resident immune and stomal cell populations. Note the absence of any organized immune aggregates in healthy skin, and (B) three example scenarios where TLSs have been observed in the skin: melanoma, cutaneous squamous cell carcinoma, and Hidradenitis suppurativa with the described localization of TLSs indicated.

The skin consists of three distinct layers: the epidermis is the external‐facing layer, with the dermis underneath and the hypodermis (or subcutaneous fat) below the dermis (Figure 3A) [123]. The epidermis primarily consists of keratinocytes (epithelial cells), which proliferate, differentiate, and cornify through the epidermal layers, eventually forming the characteristic desiccated layer on the skin surface. Epidermal maturation occurs at 34 weeks in embryonic development, prior to which common macrophage precursors seed Langerhans cells in the lower epidermal layers [124]. Langerhans cells will locally proliferate in situ into adulthood, working to survey the local environment by extending and retracting dendrites between keratinocytes without disturbing the epidermal structure [125, 126]. In response to this environmental sampling, Langerhans cells may migrate to skin‐draining lymph nodes to prime T cells and contribute to active adaptive responses [127]. Locally in the skin, Langerhans and tissue‐resident T cells are involved in the maintenance of skin homeostasis and regulation of keratinocyte turnover, differentiation, and repair [128, 129]. Resident T cells in the epidermis at steady state consist of specialized intraepithelial lymphocytes, which seed the tissue during fetal development [130]. Intraepithelial lymphocytes, many of which express a γδ TCR, are unconventional innate‐like T cells and represent one of the most abundant and evolutionarily ancient lymphocyte populations in the body. As we mature and become antigen‐experienced, areas of the epidermis will in addition harbor populations of tissue‐resident memory T cells which are conventional αβ T cells predominantly co‐expressing the CD8 receptor. Both intraepithelial lymphocytes and tissue‐resident memory T cells maintain their position in the epidermis through expression of CD103 and low‐level proliferation [131, 132, 133].

The underlying dermal layer is more diverse and consists of an array of different cell populations, including structural cells and immune cells. The primary structural component is made up of fibroblasts, which contribute to the maintenance of healthy skin structure through the production of ECM proteins, such as collagen and elastin, providing support and elasticity to the skin [134]. Human dermal fibroblasts can be divided into two classes: the reticular fibroblasts, which reside just above the hypodermis, and papillary fibroblasts, which sit just under the epidermis [135]. Reticular fibroblasts are sparsely populated throughout the thick, well‐organized matrix fibers of this region, demonstrating low‐level proliferation and high production of extracellular protein Papillary fibroblasts, meanwhile, are densely populated and carry out less protein production, instead exhibiting a Wnt signaling signature and elevated degrees of proliferation [136]. During skin aging, the papillary dermis decreases in volume, which is associated with a decrease in proliferation in the basal keratinocyte layer in the healthy aged epidermis [137].

In addition to the structural cells present, the dermis comprises a wide range of different immune cell populations. Resident immune cell populations include mast cells, which seed during fetal development, different subsets of DCs, macrophages, ILCs, conventional αβ T cells, dermal γδ T cells, and a specialized population of regulatory T cells which seed around the hair follicle perinatally [138]. Thus, healthy human skin contains a large number of resident lymphoid cells, but very few, if any, B cells [139]. Other immune cells in the dermis are transient in nature, such as monocytes and neutrophils, which pass through the lymphatic network that sits at the bottom of the dermis. While there is clearly controlled organization of the immune compartment in the skin, there are no organized lymphoid structures or aggregates in healthy skin (Figure 3A) [140].

### 4.2 TLS in Human Skin

Formation of lymphoid aggregates in the skin was first described by Streilein et al. nearly half a century ago, who called these ‘skin associated lymphatic tissue’ (SALT) [141]. It was clear, however, that formation of SALT required specific inflammatory cues, and so was subsequently referred to as ‘inducible skin associated lymphoid tissue’ or iSALT [142]. iSALT has been most commonly described in response to topical hapten exposure and predominantly consists of T cell and DC clusters. As no naïve T cells or B cells are present, these iSALT clusters have been suggested to be sites specialized for the activation of effector T cells [143, 144]. Thus, a notable difference between iSALT and TLSs is the absence of B cells in the former, with subsequent debate around the existence of proper lymphatic tissue and TLSs in the skin ongoing until recently.

#### 4.2.1 Autoimmune and Inflammatory Skin Diseases

T and B cell clusters resembling TLSs have now been identified in several inflammatory skin conditions, enabling the potential of the skin to act as a niche for local antibody production (Figure 3B). Indeed, several of the dermal conditions wherein TLS‐like structures have been identified are cutaneous autoimmune diseases. These diseases are debilitating and numerous, and yet the role of local B cells in their pathogenesis is under‐investigated. Most notably, the autoimmune skin blistering diseases, both the pemphigus and the pemphigoid groups, have been linked to TLS development. In pemphigus, specifically pemphigus vulgaris and pemphigus foliaceus, it was found that a high percentage of skin lesions contained B cells, plasma cells, HEVs, and CD21+ monocytic cells, indicating prevalent TLS development [145]. Interestingly, in chronic blisters, B cells associated with TLSs were shown to be specific for desmoglein, demonstrating the potential for local emergence of autoantibodies which may contribute to the condition [146]. In the pemphigoid group, autoreactive B cells are also accepted to be key players in the pathogenesis. Notably, in the most common subtype, autoantibodies against hemidesmosomal proteins are present, which destroy the dermo‐epidermal adhesion and clinically manifest as tense blisters, which may also develop into eczema‐like urticarial lesions, which cause intense pain and pruritus [147].

In several other non‐blistering autoimmune cutaneous diseases, B cells and TLS‐like structures have been noted in the skin and may contribute to tissue pathology. In cutaneous lupus erythematosus, high levels of B cell activating cytokines have been demonstrated in the skin, as well as B/T cell aggregates with high levels of CXCL13 and HEVs present locally [148]. Dermatomyositis is another systemic autoimmune disease that primarily affects the skin and muscles, which has also been linked with TLS development [149, 150]. Meanwhile, pyoderma gangrenosum is a rare skin condition causing painful ulcers, wherein the recent use of spatial transcriptomics has revealed a hitherto unrecognized major B cell infiltration and development of TLSs [151]. B cell depletion therapy, such as Rituximab treatment, has been demonstrated to be effective in the treatment of some of these autoantibody‐mediated cutaneous diseases, such as severe pemphigus and bullous pemphigoid [152, 153]. However, there is a large contribution of the microenvironment to the sensitivity of B cells to Rituximab, and examples showing persistence of TLSs have been shown, despite treatment with B cell depleting drugs. This demonstrates that ‘local autoimmunity’ may be more difficult to reach therapeutically [154, 155, 156].

Skin TLSs have also been identified in other inflammatory skin conditions, such as Hidradenitis Suppurativa (HS) (Figure 3B), which, although not considered to be a true autoimmune disease, has clear immunological input in pathogenesis and has some autoreactive aspects. HS is a chronic, debilitating inflammatory skin disease characterized by epithelial tunnels growing both superficially and deep into the dermis. These cause skin abscesses, pain, and scarring. HS has been linked to autoantibody formation, although the mechanisms underlying the development of an adaptive immune response in HS are not well understood. However, new work on HS has offered extensive insight into how TLSs can develop and become established in human skin. In a recent study by Yu et al. [157], TLSs were found adjacent to HS tunnels, which were enriched with proliferating T and B cells. Extensive clonal expansion of plasma cells was found at the periphery of the TLSs, producing antibodies reactive to keratinocytes. Interestingly, it was demonstrated that fibroblasts, activated by the inflammatory microenvironment, were key to the triggering of lymphoid aggregation via their production of CXCL13 and CCL19, recruiting B and T cells, respectively. CXCL13 and FDCSP (an FDC marker) were produced by fibroblasts originating from the reticular lineage, while CCL19 was produced by papillary‐lineage fibroblasts, along with increased SPARCL1 (an HEV marker) and ICAM1. This provides an interesting mechanism wherein the two fibroblast populations of the healthy human dermal skin compartment offer two separate functional requirements for TLS development and establishment. While both may be involved in immune cell recruitment, reticular fibroblasts may be able to act as FDCs, while papillary fibroblasts may differentiate into HEVs and thus be involved in the establishment of more mature TLSs. Much remains to be discovered, but the work by Yu et al. gives a fascinating glimpse into the complex regulation between immune cells and stromal cells required to re‐organize the skin to form TLS structures.

#### 4.2.2 Cancer

Cancer represents a major disruption of the structure of healthy skin. Skin cancers are primarily categorized into: (1) epithelial cancers, basal‐ or squamous cell carcinoma (SCC) where malignant epithelial cells over‐proliferate in the epidermis and invade into the lower layers of the skin disrupting normal homeostasis; (2) Merkel cell carcinoma, a rare but highly aggressive form of skin cancer initiating in specialized basal epithelial cells called Merkel cells; and (3) melanoma, derived from the malignant transformation of melanocytes and the most metastatic of skin cancers. To date, TLSs have been described in SCC, Merkel cell carcinoma, and melanoma (Figure 3B). In all three cases, the presence of TLSs has been linked to overall better patient prognosis. In cutaneous SCC, the abundance of tumor B cells and the presence of TLSs were associated with better histopathological grades, lower lympho‐vascular invasion, and overall improved survival [158, 159]. In Merkel cell carcinoma, patients with TLS‐positive tumors had significantly better prognosis than those with TLS‐negative tumors [160]. Furthermore, the TLS‐positive tumors showed significantly elevated expression of the chemokines or their receptors, CCL5, CCR2, CCR7, CXCL9, and CXCL13, and elevated expression of just two chemokines (CXCL13 and CCL5) could differentiate patients with better prognosis [161]. In melanoma, the presence of TLSs has been linked to an improvement in overall patient survival and a reduced risk of tumor recurrence [162, 163]. TLS maturation, defined by the increased presence of DCs and HEVs, has also been shown to be associated with reduced metastasis in melanoma [164]. Furthermore, a study on the efficacy of immune checkpoint blockade in melanoma demonstrated that B cell signatures and the presence of TLSs were enriched in patients who responded to immune checkpoint blockade, compared to nonresponding patients. It was also shown that the B cells found in immune checkpoint blockade responders were switched memory cells and were clonally expanded, demonstrating an intriguing potential for the involvement of local B cells and TLSs in the response to immune checkpoint blockade in cancer [165].

Therefore, therapeutics encouraging the induction of skin cancer‐associated TLSs is an attractive concept to boost anti‐cancer immune responses as well as immunotherapy. However, our understanding of how TLSs are initiated in cancer is still limited. It seems clear that priming of local tissue stromal cells, especially fibroblasts, is a crucial step in the formation of skin TLSs. It has been suggested that differentiation of a population of podoplanin‐positive stromal cells into a network of ‘immunofibroblasts’ is key to support the earliest phases of TLS establishment. The priming of fibroblasts can then initiate a fibrous reticulum scaffold, which may provide space for TLS formation [166]. These early fibroblast priming events precede lymphocyte infiltration into the tissue and must thus rely on local inflammatory cues for initiation. In mucosal sites, such as the salivary gland, this initial ‘immunofibroblast’ priming has been shown to be mediated by paracrine and autocrine IL‐13 [167]. It is tempting to speculate that a similar reliance on IL‐13 for early priming and remodeling could be true in the skin, where IL‐13 is prevalent following cancer‐inducing tissue damage (UV‐irradiation, exposure to topical carcinogens), and is produced particularly by specialized tissue‐resident IELs [168]. In the skin, the presence of IL‐13 ultimately protects against cSCC development in mouse models [168]. Local skin‐induced cytokines also appear to be key to activation of both the reticular and papillary fibroblasts needed to initiate TLSs in HS. Here, TNF‐α was shown as an important mediator of fibroblast activation and early blockade of TNF‐α suppressed lymphoid aggregate initiation [157].

In cancer, the relationship between these early ‘mmunofibroblasts’, generally seen as aiding in anti‐tumor immunity, and cancer‐associated fibroblasts, which are generally thought to promote cancer progression, is not clear. It is evident, however, that fibroblasts are a central component of the tumor microenvironment and can profoundly influence the behavior of both the surrounding immune cells and cancer cells. Dermal fibroblasts, although seemingly positively involved in TLS development in HS, have also been implicated as pro‐carcinogenic cancer‐associated fibroblasts. Interestingly, reticular fibroblasts have been demonstrated to be more pro‐carcinogenic than papillary fibroblasts, as organotypic models of skin cancer show a higher degree of epithelial invasion and expression of cancer‐associated fibroblast markers when reticular fibroblasts are used, compared with papillary fibroblast‐i equivalents [169]. Additionally, reticular fibroblasts become positive for αSMA more rapidly in response to stimuli than papillary fibroblasts in culture [170]. The exact developmental pathway of these cells into cancer‐associated fibroblasts is not currently known, but it is clear that these fibroblasts show a high degree of versatility and plasticity in a context‐dependent manner, and thus local microenvironmental cues are key. Furthermore, a tumor that originates in the epidermis, such as cSCC or melanoma, will encounter papillary fibroblasts more immediately than reticular fibroblasts once it invades the dermis and lower skin layers. Whether the tumor would transverse the papillary layer to elicit cancer‐associated fibroblast phenotypes from reticular fibroblasts, or whether papillary fibroblasts migrate alongside the tumor front and develop more reticular fibroblast‐like phenotypes, as they have been shown to do in culture [171], remains to be determined.

### 4.3 Mouse Models of Skin TLSs

A limitation to the mechanistic understanding of the development of TLSs in skin has thus far been the difficulty of inducing these in mouse models. Previously, dermal inflammatory conditions and cancers have consistently appeared to be incapable of developing TLS structures in mouse skin, while they appear quite readily in other tissues [172, 173]. While iSALT and T cell aggregates are visible in contact dermatitis models, these were devoid of B cell structures [142]. In melanoma models, some B cells could be detected following tumor induction, but these were not GC‐like, nor aggregated into TLS‐like structures [174].

To date, two approaches have successfully elicited B cell aggregates that resembled TLSs in mouse skin cancer models. The first, surprisingly, involved the severing of sensory neurons prior to melanoma establishment. Both surgical and chemical cutaneous denervation was shown to result in the formation of classical TLSs with mature HEVs, which augmented melanoma‐specific immune responses and were associated with favorable outcomes [175]. Furthermore, tumors arising in denervated skin had enhanced lymphoid and myeloid cell recruitment and an expanded B cell repertoire, demonstrating that in this model, cutaneous sensory nerves impeded TLS formation and anti‐tumor immunity. The second approach involved seeding fibroblasts from an established intraperitoneal tumor into the mouse dermis. Only when these intraperitoneal tumor‐derived fibroblasts were seeded alongside a melanoma cell line would tumor‐associated TLSs develop [166]. This clearly highlights the importance of activated fibroblasts in the development of lymphoid aggregates.

Why mouse skin fibroblasts appear less prone than humans to develop into ‘immunofibroblasts’ and support TLS development is not clear. However, it must be noted that there are overt differences between mouse and human skin, which represent both an obstacle and also an opportunity to understand the underlying requirements for TLS initiation. Mouse skin has a thinner epidermal layer and increased follicle density, but there are also key differences in the fibroblast composition [176, 177]. In mouse dermis, the papillary layer is thinner than that in humans, with fewer papillary fibroblasts in residence [136, 178]. It is possible that the reduction in this compartment is the limiting factor in TLS development, which is supported by data showing that TLSs could be induced in the skin *de novo* when this fibroblast pool was enriched with cancer‐associated fibroblasts from a separate tumor [166]. It is also unclear whether papillary fibroblasts from mouse skin can adopt the TLS‐supportive SPARCL1‐ and ICAM‐1‐expressing phenotype that has been shown in humans [157]. It has been demonstrated previously, however, that neonatal dermal fibroblasts are able to adopt an LTo‐like phenotype, which included LTβR expression, in response to TNF‐α stimulation, but that this was lost once the mouse skin reached maturity [179].

One exception to the general difficulty in inducing TLSs in mouse skin is a new study showing that topical skin infection with the human commensal *Staphylococcus epidermidis* can promote the formation of TLSs in the dermis, including the induction of functional GC‐like structures [180]. The skin B cells were highly expanded clones that were predominantly unique to the skin compartment and were able to support skin‐autonomous antibody production. It is currently unclear exactly how *S. epidermidis* induced these structural changes in the mouse dermis, but the TLS structures were located around the hair follicle, which is the primary site for microbial colonization, and Langerhans cells were involved in promoting T cell help for the induction of skin humoral immunity.

While mouse skin is clearly notably different in several aspects from human skin, there are also many similarities, and the immunological composition is generally comparable. It may well be that mouse models represent an opportunity for novel tool development to further our understanding of TLS development and the contribution of dermal fibroblasts to these immune cell aggregates, as well as their function in immune homeostasis.

## 5 Conclusion

TLS are dynamic and highly organized aggregates of lymphocytes and stromal cells that form in response to persistent inflammatory signals and play a variety of roles across different tissues and diseases. Although TLSs share anatomical and functional characteristics with SLOs, their unique development within inflamed tissues highlights their importance in local immune responses. The remodeling of fibroblasts to acquire an SLO‐like phenotype expressing CCL19/21, CXCL13, and/or CXCL12 is central to TLS formation across tissues and disease contexts; however, there is variation in the inflammatory and immune cell signals required for fibroblast remodeling. Fibroblasts that are permissive to remodeling and differentiation are exemplified by the skin, where the lack of pre‐clinical models may relate to the stark difference between human and mouse skin fibroblasts. Recent advances have highlighted the potential of TLSs. In autoimmune disease, TLSs heighten inflammation and are strongly associated with disease severity. In cancer, TLSs are strongly associated with better outcomes and improved responses to immune checkpoint blockade, although whether the immune checkpoint blockade response is specific to TLSs is not clear—the presence of TLSs may be symptomatic of increased systemic immune responses. This question aside, there is clear evidence that TLSs can support functional immune responses that generate high‐quality responses, albeit with a delayed kinetic for formation; the data are clear that the presence of TLSs potentiates local immunity. Thus, TLSs have potential as therapeutic targets. In cancer, TLSs offer promise not only as a prognostic tool but also as a therapeutic target to promote anti‐tumor immunity; conversely, disruption of TLSs in autoimmune diseases may alleviate immune‐driven disease.

## Conflicts of Interest

A. C. and R. N. are employees of GSK and hold stocks and shares in the company. J. Shelley, J. Strid, and A. E. D. declare no competing interests.

## Data Availability

Data sharing not applicable to this article as no datasets were generated or analyzed during the current study.
